# Effort-Reward Imbalance: A Risk Factor for Exposure to Workplace Bullying

**DOI:** 10.3389/fpsyg.2019.00386

**Published:** 2019-02-25

**Authors:** Guy Notelaers, Maria Törnroos, Denise Salin

**Affiliations:** ^1^Department of Psychosocial Science, Faculty of Psychology, University of Bergen, Bergen, Norway; ^2^Department of Management and Organization, Hanken School of Economics, Helsinki, Finland

**Keywords:** workplace bullying, effort-reward imbalance, injustice, fairness, strain, stress, work environment

## Abstract

Previous research shows that work environment factors are important antecedents of workplace bullying (WB), because of the stress they may induce. While previous studies have typically used Karasek’s Job Demand-Control model or the Demands-Resources model, the present study investigates whether another important occupational stress model, that is the Effort-Reward Imbalance model, is also associated to WB. A survey study in 19 Belgian organizations (*n* = 5727) confirmed that employees experiencing an imbalance between efforts and reward were more likely to be targets of exposure to bullying. In line with previous research, this study illustrates that stressful situations increase the risk of exposure to WB. It shows that the perceived incongruence between effort and reward may increase employee vulnerability to bullying. The perceived injustice may lead employees to engage in norm-breaking behavior and also signal low social standing to others, thereby potentially eliciting negative behaviors from others.

## Introduction

Prominent scholars agree that bullying at work means harassing, offending and socially excluding someone over a longer period of time (Einarsen et al., 2011). Being repeatedly and systematically exposed to negative behaviors is the common denominator of definitions of workplace bullying (WB) (Notelaers and Einarsen, 2013; Notelaers et al., 2013). Research has shown that WB threatens employees’ mental and physical well-being at work (Nielsen and Einarsen, 2012).

During the last decade, researchers have started to look for theoretical frameworks explaining more specifically why different environmental features are associated with a higher risk of bullying, providing us with a deeper understanding of the WB phenomenon (e.g., De Cuyper et al., 2009). For instance, researchers have used different stress models, such as the Job Demand-Control model (Karasek, 1979), the Job Demand-Resources model (Bakker and Demerouti, 2007), and role stress (Beehr and Glazer, 2005), showing that stress makes employees vulnerable to WB (Baillien et al., 2011a,b; Notelaers et al., 2013; Reknes et al., 2014).

Yet, stress stemming from high job demands and lack of control or support or resources is not the only path to vulnerability. Experiences of injustice have also been reported to be a severe stressor in the workplace (Greenberg, 2006). In line with the idea that work-related stress may induce bullying and that perceptions of injustice are a central stressor, the present study aligns with Guglielmi et al. (2018) to investigate whether the Effort-Reward Imbalance (ERI) Model (Siegrist, 1996), is valuable in understanding reports of exposure to WB.

### The Effort-Reward Imbalance Model and Bullying

In the ERI model, work-related stress is conceptualized as lack of fairness of the reciprocity of efforts expended and reward received at work (Siegrist, 1996; Siegrist et al., 2004). Thus, the model is concerned with social reciprocity and reflects distributive justice at work (Siegrist et al., 2004). In the model, effort means the demands and obligations the employee is faced with, and reward the money, esteem, and career opportunities (or job security) the employee expects in return, not only from the employer but also from society at large (Siegrist, 1996).

The ERI hypothesis states, that it is the combination of high effort and low reward (effort-reward imbalance) that increases the risk of poor health over the risks associated with each of the components alone (van Vegchel et al., 2005). The experience of a lack of reciprocity creates negative feelings in an employee (Siegrist, 1996). In the long run, this increases illness susceptibility as a result of continued strain reactions in the autonomic nervous system (Siegrist, 2005).

In analogy with other stress models, we argue that ERI may also be a risk factor for bullying. Previous research highlights that work stressors may increase the risk of exposure to bullying through several different mechanisms. For example, inefficient coping with stress and frustration may lead employees to behave in ways that violate norms (Baillien et al., 2009). This can include, for example, decreasing the level of work efforts, persistent complaining, and withdrawing from social interaction. Such behaviors may, in turn, elicit retaliatory action and victimization from colleagues and superiors, trying to reign in or punish the norm-breaking employee (Neuman and Baron, 2003). Also, stress resulting from effort-reward-imbalance may, as discussed above, result in mental health problems (Bonde, 2008), which again have been shown to increase the risk of being subject to subsequent exposure to bullying (Nielsen et al., 2012).

In spite of the similarities, different stress models are not interchangeable but complementary, and reflect slightly different aspects of the psychosocial work environment (Siegrist et al., 2004; Tsutsumi and Kawakami, 2004). Where, for example, the Job Demand-Control model highlights task-level control, the ERI model puts the spotlight on the reward the employee receives (Siegrist et al., 2004). According to the ERI model, reward reflects distributive justice (Siegrist, 2001), which refers to how the employee perceives the fairness of the outcomes relative to the contribution (Colquitt, 2001). Being rewarded for one’s effort with lack of career opportunities, meager financial growth prospects, or lack of recognition by both colleagues and leaders may be seen as unfair. Furthermore, when an employee is under-rewarded with respect to their efforts, this may also signal to colleagues and superiors that the employee has a low social standing and little support from management. As a power imbalance between perpetrator and victim is an important part of the definition of bullying (Einarsen et al., 2011), low (social) power can be seen as a clear risk factor (Salin, 2003).

Hypothesis: Employees reporting a higher degree of imbalance between efforts and rewards (i.e. who are under-rewarded in comparison to their efforts) have a higher likelihood to be a target of bullying.

## Methods

### Sample

This study uses data – which is available upon request – from employees in 19 Belgian organizations. The questionnaire data were collected between 2003 and 2006, as part of studies to assess and prevent psychosocial risk factors at work. The mean response rate in these surveys was 70%. The final sample for this study consisted of 5727 respondents.

A small majority of the respondents (56%) were male. Mean age was 41 years (*SD* = 10) and mean tenure was 12 years (*SD* = 11). Almost 9.8% were blue collar workers, 30.7% white collar workers, 0.3% nurses or social workers, 7.9% managers, 10.9% top managers, and 40.9% public servants. Twenty-one percent held supervisory responsibilities. Only 1.2% worked in the manufacturing industry, 48.3% in the service sector, 30.9% in governmental services, and 19% in the public health sector. Eighty percent had a permanent contract, 13% had a temporary contract whereas 7% had another type of contract.

### Ethics Statement

According to the relevant institutional and national guidelines, no review and approval from an ethical board was required at the time of data collection. Similarly, as the study did not contain any health related data, explicit informed consent was not needed according to the institutional and national standards of Belgium in 2006. However, respondents were informed about the goal of the project, and that their data were used for benchmarking purposes and for scientific research. Participation was voluntary, and anonymity was guaranteed. Person data was made anonymous which is also in line with the current European General Data Protection Act.

### Measurements

Workplace bullying was measured with a 16-item version of the NAQ (Notelaers et al., 2011, 2013). We asked respondents how often they had been subjected to the 16 negative social behaviors during the last six months. The response categories were “never,” “now and then,” “once a month,” and “once a week or more often.” This measure had a satisfactory reliability (α = 0.89).

Because the questionnaire did not operationalize effort and reward using Siegrist’s (1996) measure we operationalized them using the Questionnaire on the Experience and Evaluation of Work (van Veldhoven and Meijman, 1994). Effort was measured with 5 items. A sample item was as follows: “Do you have to work very fast?” Reward was measured with 7 items from the same questionnaire. Items included for example: “Do you think you are paid enough for the work that you do?” The response categories for the Effort and Reward items were “always,” “often,” “sometimes,” and “never.”

A principal component analysis in SPSS confirmed that the 12 items for effort and reward loaded onto two different latent components. Importantly, none of the indicators loaded less than the threshold of 0.4 (Tabachnick and Fidell, 2014) on its own factor and most cross loadings were lower than 0.2. Only one cross loading was 0.26 which still is lower than the 0.3 threshold (Tabachnick and Fidell, 2014). The Cronbach α of Effort was 0.68 whereas that of Reward was 0.70 which is satisfactory (Eggen and Sanders, 1993; Aron et al., 2013). The ERI ratio was calculated as the ratio 5/7 ^∗^ Effort/Reward as suggested by Siegrist et al. (2004, p. 1487).

### Procedure

This study employed a secondary analysis of existing data that was partially published earlier (Notelaers et al., 2010, 2013) to identify bullying using latent class cluster modeling. As a result, the database contained the conditional probabilities to belong to a certain latent class cluster. Six clusters could be identified: not bullied, experienced limited work criticism, had limited negative encounters, occasionally bullied, work related bullying, and victim of bullying (cf. Einarsen et al., 2009; Notelaers et al., 2011 for a similar methodology). To test our hypothesis, the probability that a respondent was a victim of bullying, that is having the highest likelihood to be weekly or more often subjected to a wide range of systematic negative behaviors, was the dependent variable. The latter aligns in the strict sense with the definition of bullying (Einarsen et al., 2009; Notelaers et al., 2011).

## Results

Table 1 portrays the descriptive statistics of the current study.

**Table 1:** Descriptive statistics.

	Mean	*SD*	1	2	3	4
(1) Gender			1.000			
(2) Supervisory position			0.164^∗∗^	1.000		
(3) Effort	2.433	0.53	0.030^∗^	0.199^∗∗^	1.000	
(4) Reward	2.432	0.51	0.066^∗∗^	0.116^∗∗^	-0.178^∗∗^	1.000
(5) Probability to be a victim of bullying	0.0356	0.12	-0.013	-0.021	0.189^∗∗^	-0.452^∗∗^


*^∗^p < 0.05, ^∗∗^p < 0.01, ^∗∗∗^p < 0.001; Correlations are spearman rho correlation coefficients.*

In the zero-inflated latent class regression analysis in Latent Gold 5.0 (Vermunt and Magidson, 2015), the independent variables were regressed on the dependent variable in three steps: (1) control variables, (2) effort and reward, and (3) ERI. The results for the regression models are shown in Table 2. The main result was, however, that high ERI is positively related with the probability to be a target of bullying.

**Table 2:** Unstandardized regression estimates for the association between effort-reward imbalance and exposure to bullying.

	Step 1	Step 2	Step 3
Intercept	0.024	0.137	-0.184
Supervisory responsibilities (yes = 0, no = 1)	0.011^∗∗∗^	0.009^∗∗∗^	0.009^∗∗^
Gender (male = 0, female = 1)	-0.002	-0.004	-0.003
Occupational status			
Blue collar	0.036^∗∗∗^	0.032^∗∗∗^	0.027^∗∗^
White collar	-0.011	-0.013	-0.014
Nurses, assistants	-0.024	-0.018	-0.014
Public servant	0.020^∗∗∗^	0.019^∗∗^	0.015^∗^
Manager	-0.011	-0.011	-0.009
Top manager	-0.010	-0.008	-0.007
Effort		0.024^∗∗∗^	-0.101^∗∗∗^
Reward		-0.070^∗∗∗^	0.064^∗∗∗^
Effort-reward imbalance			0.396^∗∗∗^
Error Variances	0.031^∗∗∗^	0.029^∗∗∗^	0.028^∗∗∗^
**Fit statistics**			
CAIC(LL)	-3540.97	-3706.02	-3884.13
BIC(LL)	-3550.97	-3718.02	-3897.13
R^2^	0.009	0.054	0.082


*^∗^p < 0.05; ^∗∗^p < 0.01; ^∗∗∗^p < 0.001; CAIC(LL), consistent akaike information criteria; BIC(LL), Bayesian information criteria (n = 5727).*

## Discussion

Our study aimed to advance insights into the work environment hypothesis by investigating the role of a well-established theoretical stress framework (ERI), so far only investigated once in bullying research (Guglielmi et al., 2018). Our research advanced research by employing a large sample of employees, employed in 19 organizations across different sectors. That our research corroborates previous research notwithstanding its use of an alternative operationalization of effort and reward is a testimonial for the importance of the ERI model to understand exposure to WB.

In line with previous findings (Guglielmi et al., 2018) we find that an imbalance between effort and reward was associated with an increase in the likelihood to be a target of exposure to WB. This also supports earlier findings, suggesting that work-related factors may contribute to the risk of bullying through a stress process (Hoel and Salin, 2003; Baillien et al., 2009). Based on previous research we believe there are several mechanisms possibly explaining this association: unfairly treated and frustrated employees may violate norms and are therefore punished (Neuman and Baron, 2003), and under-rewarded employees may further be particularly vulnerable because of perceived lower social power or because they experience mental distress and depletion of energy (e.g., Nielsen et al., 2012).

### Limitations, Suggestions for Future Research and Implications

The first limitation of the present study is its cross-sectional research design, which does not allow for any claims on causality. Another possible limitation is that we did not use the original measures to operationalize efforts and reward. Yet both the exploratory factor and the reliability analyses support the presence of two reliable factors that we labeled as effort and reward. Moreover, the replication of earlier findings by using an alternative operationalization of ERI supports the validity of the latter to understand exposure to WB.

Because the current results are promising and corroborate earlier research (Guglielmi et al., 2018), we suggest that future research not only replicates, but also extends our study and Guglielmi’s study explicitly studying not only perceptions of injustice as a mediation mechanism but also strain. In addition, future research may also investigate overcommitment as a moderator, as suggested in Siegrist’s (1996) original model. In the case of bullying, overcommitment may not only make employees more prone to experience ERI as more stressful and unfair, but they might also experience more bullying. Finally, as the same stressors may increase both the risk of exposure to bullying and enactment of bullying (Baillien et al., 2009), future research may further study if ERI also increases the enactment of WB.

The finding that ERI contributes to the experience of bullying has important consequences for the compensation functions of organizations. It points to the importance of increasing fairness in compensation if organizations want to reduce the risk of bullying.

## Author Contributions

GN collected and analyzed the data, and initiated and wrote the manuscript. MT contributed with the ERI model, and analysis and discussion. DS contributed to the entire manuscript, and added or better deepened the fairness issue we are dealing with.

## Conflict of Interest Statement

The authors declare that the research was conducted in the absence of any commercial or financial relationships that could be construed as a potential conflict of interest.
